# Mammography: a review of records in the Department of Radiology at a National Referral Hospital in Uganda

**DOI:** 10.11604/pamj.2014.18.89.3237

**Published:** 2014-05-26

**Authors:** Elsie Kiguli-Malwadde, Roy Gonzaga Mubuuke, Sam Bugeza, Brian Mutungi

**Affiliations:** 1 Department of Radiology, Mulago Hospital, Kampala, Uganda

**Keywords:** Breast, mammography, breast imaging, radiology, Uganda

## Abstract

**Introduction:**

Breast cancer is one of the leading cancers amongst women world-wide. Although mortality has been reduced and survival rates increased in developed countries, mortality rates from breast cancer are still a major health challenge for many developing countries. In Uganda, there are no screening programmes and in many cases mammography is used for diagnostic purposes. The purpose of this study was to describe the clinical presentations and mammographic breast density patterns amongst women that presented to the radiology department for mammography at a national referral hospital.

**Methods:**

This was a retrospective study carried out at Mulago Hospital in Uganda between January 2011 and January 2012. Records for patients who had mammography during this period were reviewed.

**Results:**

The total number of patients was 382 with a mean age of 46 years. Majority presented with breast pain and masses. Mammograms done were normal in majority of the women with fatty breast density dominating. In Uganda, mammography was mainly performed for diagnostic purposes.

**Conclusion:**

There is no mammography screening programme in Uganda and many women cannot access the service due to its limited availability and costs despite its significance in breast cancer management. There is therefore need for governments in Uganda, but in other areas as well to support regular mammography screening as a way of reducing mortality from breast cancer.

## Introduction

Breast cancer is the third commonest cancer in women in Uganda following Kaposi sarcoma and cervical cancer [1]. A previous study conducted in Uganda reported breast cancer incidence at 22: 100,000 with a five year survival rate of 56% [2]. Breast cancer has also been reported as being one of the most frequent cancers of women in Asia [3]. Although breast cancer incidence rates showed signs of decrease in Canada in 2002, later studies conducted demonstrated a renewed increase in 2004/2005 [4].

In the United States, a study from the Kaiser Permanente Northwest healthcare system reported a decrease in breast cancer incidence from 2000 to 2006 [5]. However, closer scrutiny of data for each individual year reveals that this decrease stagnated around 2004 in older women and rates increased in women aged 45-59 through up to 2006 [5]. In the UK, survival rates have continued to increase but breast cancer still remains a priority health challenge [6]. Breast cancer therefore remains one of the most significant metastasizing tumour in women with an estimated 1.4 million women having been diagnosed in 2008, of whom 460,000 dying of the disease with majority from Africa [7]. In 2008, there were an estimated 30,000 new breast cancer cases in West Africa and 16,000 deaths, while Eastern Africa had an estimated 18,000 new cases and 10,000 deaths [7].

Therefore, early diagnosis of breast cancer is the most significant factor in increasing survival rate and reducing mortality [8]. Although Poplack et al. [9] reported that breast biopsies can reliably distinguish between malignant and benign abnormalities in more than 75% of women, Houssami et al. [10] observe that imaging unarguably plays an equally vital role many times preceding the clinical decisions to perform biopsies. For instance, regular mammographic screening has been reported to decrease breast cancer mortality by 15%-20% [11, 12]. Consequently, regular mammographic screening of women has been adopted in a number of countries [12].

Breast cancer mortality rates have generally decreased in the developed world over the years with increased case survival rates [13, 14]. On the contrary, mortality rates in developing countries are still significantly high. Closely interrogating reasons for this difference, points to the easy availability and accessibility of mammography screening programmes for early detection and thus treatment of breast cancer in the developed world. Uganda like many developing countries has no such regular and routine mammography screening programmes.

The Uganda Breast cancer guidelines published in 2007 [1] recognize the importance of mammography in assessing breast lesions. Although these guidelines recognize and recommend the use of mammography, it is not feasible for regular mass screening in Uganda, as there are a limited number of health facilities with mammography services. Additionally, accessibility and affordability are hindered by the lack of regular government-funded screening initiatives, an observation that has also been reported in other developing countries [8]. In Uganda therefore, mammography has been mainly used as a diagnostic tool alongside Ultrasound as a complimentary imaging modality to not only characterize breast lesions, but also in the breast assessment of non-symptomatic ladies of reproductive age. Thus, at Mulago hospital where this study took place, mammography has been largely utilized for purposes of diagnosis in already symptomatic cases. Mulago being a national referral hospital, the largest number of mammography investigations are carried out in the hospital‘s radiology department. The purpose of this retrospective study therefore, was to investigate the demographics, clinical presentations, mammographic patterns and findings of breast lesions amongst women presenting for mammography at Mulago hospital as well as their clinical presentations.

## Methods

**Study setting**: The study was conducted at Mulago National Referral Hospital in Uganda. Mulago Hospital is also the teaching hospital for Makerere University. It is a 1500 bed hospital. It serves the Uganda population which is 30 million people but also gets patients from surrounding countries like Rwanda, Congo and South Sudan. It is the only public hospital with a mammography machine in Uganda.

**Purpose of the study**: The objective of the study was to describe the demographic characteristics clinical presentation, mammographic findings, density patterns of patients presenting for mammography at Mulago Hospital also to describe the mammographic pattern of breast diseases at Mulago Hospital

**Study design**: This was a retrospective study. Records of all patients that had mammography in the Department of Radiology at Mulago Hospital from January 2011- January 2012 were reviewed. Using a pre tested data collection instrument, data was collected by a research assistant. The radiologists reviewed the mammograms and reports.

**Data analysis**: Data was entered into the Epi-Info statistical package and later analyzed using STATA. Descriptive statistics were compiled for patient demographics, patients’ presentation, mammographic findings and breast density.

**Ethical issues**: Permission to carry out the study was granted by the Mulago Hospital Research and ethics committee. No patient or any forms of identifiers were retrieved from the records to include in the data. Raw data was entered into a private computer accessed only the researchers and protected by a password.

## Results

**Figure 1:**
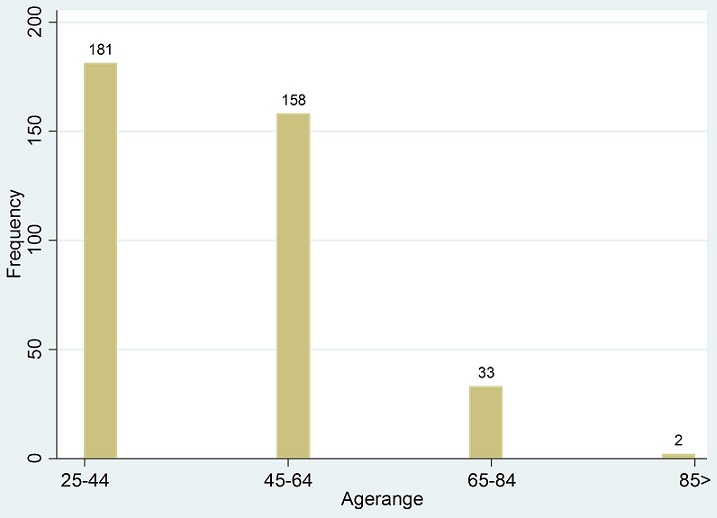
Age range of the patients

The total number of patients was 382, of whom 378(98.9%) were female and only 4(1.1%) male. Although the majority came from Buganda, their tribes were quite diverse with patients from Ateso, Banyoro, Banyankole and many others. Their ages ranged from 25 to 88 with a mean age of 46 (Figure 1). The average number of pregnancies per woman was 5, with the least being 0 and the most 15. The average age at menarche was 14, with the youngest at 9 and oldest at 18 years. Most of them had their menopausal status pre-menopausal (54.5%), post (39%) and not indicated (6.5%)

**Figure 2:**
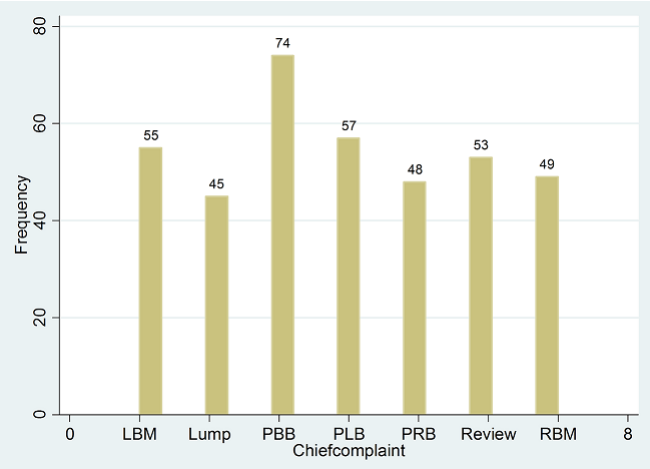
Chief complaint

**Figure 3:**
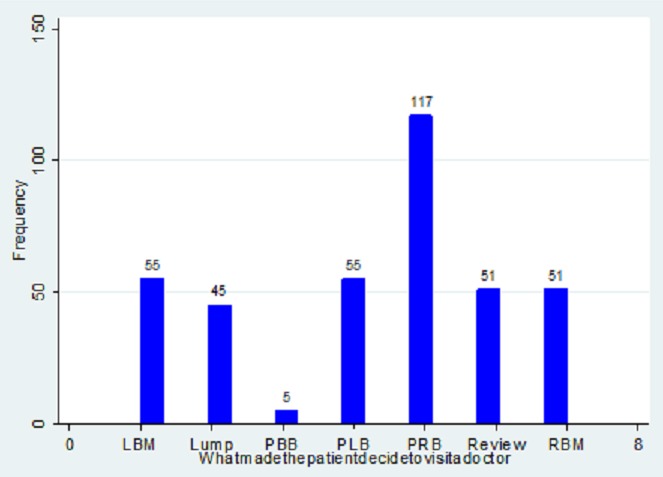
Different reasons for visiting the doctor

**Figure 4:**
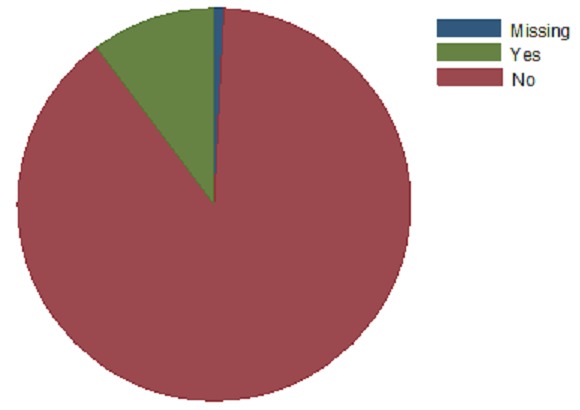
Family history of cancer

The main chief complaint were pain in either breast or both breasts (42%), breast mass (39%), Review of previous complaints (14%) (Figure 2). Key for chief complaint and reason for visiting the doctor included: LBM-Left breast mass; RBM- Right breast mass; PBB-Pain in both breasts; PLB-Pain in left breast. 148(39%) patients had lumps/mass while 222(58%) did not report lumps/masses in their breasts. 305(79%) patients reported discomfort, pain or soreness, while 377(98%) claimed the pain was not related to menstrual period (Figure 3). 80(21%) reported a discharge from the nipple with only 15(4%) claiming the discharge was bloody. 26(7%) reported skin or nipple retraction. 51(14%) had had breast biopsy or surgery. 39(10%) had a family history of breast cancer, of which 20(5%) from a 1st degree relative and 17(4%) from a 2nd degree relative (Figure 4).

**Figure 5:**
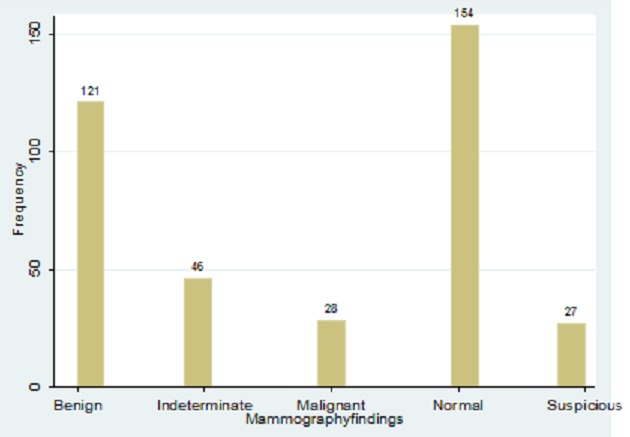
Mammography findings

**Figure 6:**
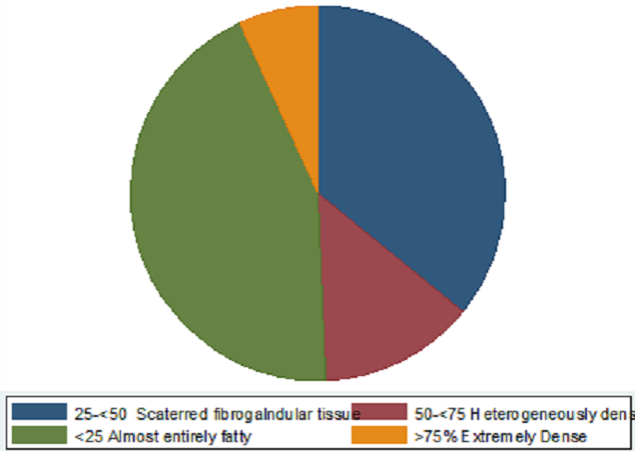
Breast density category

The Mammography findings were as follows: benign (32%), Indeterminate (12%), Malignant (7%), Normal (41%), and Suspicious (7%) (Figure 5). Breast density category findings included 25-75% Extremely Dense (7%) (Figure 6)

## Discussion

This study investigated mammographic findings and clinical presentation of women presenting in the Radiology Department for mammography. Mammography is the preferred examination of choice for the breast especially in women over 40 years for purposes of breast cancer diagnosis [15]. Although breast cancer is one of the commonest cancers in women, early diagnosis by mammography can reduce mortality. However, from this study, most patients visited the hospital after experiencing symptoms thereby making the mammogram requested a diagnostic rather than a screening investigation.

Although the Ugandan breast cancer guidelines recommend mammography screening for women of 40 years and above [1], women in this study above 40 years only reported to hospital after observing lumps, pain or discharge from the breasts. This is due to the fact that in Uganda, there is no mammography screening programme for breast cancer and as such, women only report for mammography when it is requested for by doctors for diagnostic purposes only. Unfortunately, some of the women present late after the cancer has spread yet it could have been detected during routine screening procedures [2].

The cost of routine screening programmes, together with limited access to mammography services worsens the situation. Uganda like many other developing countries grapples with limited resources to set up routine screening programs. Most health facilities in Uganda lack mammography equipment and expertise to carry out the exercise. The equipment is only available in referral hospitals meaning that only referred patients can undergo mammography. Unfortunately, by the time referrals are made, most breast cancers have passed their initial primary stages. This partly explains the high mortality rates in Uganda, despite the reduction in mortality in other countries. Rural women who need to travel long distances to access mammography are mostly affected. Sometimes such women lack money to pay for the service, a situation that is common in other developing countries without mammography screening programmes. This possibly explains the low number of women from this study (382) who had mammography in the entire year at a national referral hospital. Another plausible explanation is that mammography is not done because women with advanced stages of cancer with no need to perform a mammogram. It could also imply that clinicians are not referring patients for mammography and they need more awareness about its value or availability.

Majority of the patients complained of pain in the breasts followed by breast lumps as the main reasons for visiting the hospital and doing a mammogram, the highest incidence of these symptoms being observed amongst women in the reproductive age (25-44 years). This finding is comparable to previous studies conducted to assess breast masses [16]. It therefore appears that breast masses together with pain are a common clinical presentation of women presenting for mammography at this hospital. Mammography in this case is utilized as a diagnostic tool to characterize these masses rather than as a screening procedure.

In this study, a significant number of women had other key clinical symptoms that included nipple discharge that was sometimes bloody as well nipple retraction. These have been described in previous studies as key indicators of suspicious malignant processes occurring within the breasts [17]. Additionally, family history of breast cancer was a significant finding within this study. Lee et al. [17] reported that family history of breast cancer was a major risk factor for breast cancer and if taken into consideration, many breast cancers would be determined in their early stages if mammography screening is done. However, like already discussed, majority of the women in Uganda present to hospitals late after observing symptoms, a time when potential breast cancer has spread.

Mammograhic findings from this study indicated that majority of the women had normal breasts followed by benign, indeterminate, suspicious and malignant masses respectively. Majority of women in this study belonged to the reproductive age group. Therefore, the pain observed could have been due to mastalgia associated with physiological hormonal changes within the body and hence there were no associated abnormalities. The fact that women can feel breast pain due to hormonal changes of the menstrual cycle has been documented [18]. This might explain the normal mammographic findings despite the presence of breast pain. However, mammography also revealed that a significant number of them had a variety of masses within the breasts. The superiority of mammography to distinguish between benign and malignant masses has been emphasized [19]. A sensitivity of between 63% and 98% has been reported [20]. In line with these previous studies, this study also showed that mammography can characterize breast masses as either benign or malignant.

However, Robertson [21] cautions that accurately characterizing masses as either benign or malignant on mammography largely depends on the density of breast tissues. Although this study showed that majority of women had almost entirely fatty breast tissue, a significant number of women had densities ranging from heterogeneously dense to extremely dense (BI-RADS Categories III andIV) with a combined percentage of more than 15%. This is similar to what was reported by Galukande et al [22] that most women (67.9%) in a group studied at the same hospital had had scattered fibro glandular or fat densities (Grades I & II). Having dense breast tissue has been reported to be a risk factor for some breast malignancies [23]. Additionally, dense breasts significantly attenuate the X-ray beam reducing image quality and subsequently making visualization of subtle breast lesions a challenge and lowers overall sensitivity of mammography [24]. In previous studies, the sensitivity of mammography in dense breasts has been reported to be as low as 30% [15, 19]. This possibly explains the significant percentage of findings in this study that were simply labeled indeterminate and suspicious. In such situations of dense breast tissue, mammography becomes equivocal in accurately identifying and characterizing masses, requiring follow up with histo-pathological investigations.

Since this study was retrospective in nature, it was not possible to correlate mammography findings with with histo-pathological investigations, a limitation of the study. This also indicated a weakness in the hospital health information systems management. At this hospital results and records for patients are not linked between departments so retrospective studies are difficult. Although a previous study has been conducted with Ugandan women assessing the diagnostic accuracy of ultrasound with breast masses [25], more longitudinal observational and correlational studies are recommended involving mammography, ultrasound and histo-pathology. However, this study provides useful data regarding the significant role of mammography in the assessment of breast lesions.

## Conclusion

This study demonstrates that mammography in Uganda is only used for diagnostic purposes rather than screening and only a small number of women have access. Most women who present for mammography are less than 45 years of age, with an average parity of 5 and their main chief complaints are pain and breast mass. Most have low density breasts. Despite the fact that most women had normal results, a significant number had results that suggested malignancy. Therefore mammography remains an important screening and diagnostic investigation that should be promoted amongst women not only in Uganda, but in other areas as well. It is suggested that health policies should be formulated to ensure that regular and easily accessible mammographic screening programmes are in place if morbidity and mortality from breast cancer is to be significantly reduced.
